# Wait times for scheduling appointments with hospital affiliated dermatologists in New York City

**DOI:** 10.1007/s00403-024-03249-w

**Published:** 2024-08-17

**Authors:** Corey H. Basch, Grace C. Hillyer, Bailey Gold, Charles E. Basch

**Affiliations:** 1 Department of Public Health, William Paterson University, University Hall, Wayne, NJ 07470 USA; 2 Department of Epidemiology, Mailman School of Public Health, Columbia University, New York, NY USA; 3 Department of Health and Behavior Studies, Teachers College, Columbia University, New York, NY USA

**Keywords:** Appointment scheduling, Timeliness wait time, Dermatologist

## Abstract

Patients’ experience accessing dermatologic care is understudied. The purpose of this cross-sectional study was to examine current wait times for new patients to receive dermatological care in NYC. Websites at 58 accredited private and public hospitals in the five boroughs of NYC were reviewed to identify dermatology practices. Office telephone numbers listed on each website were called to collect information pertaining to whether the physician was accepting new patients, type of insurance accepted (public, private, both, or none), and the number of days until a new patient could be seen for an appointment. Data pertaining to the time kept on hold and availability of web-based booking were also collected. Mean waiting time for an appointment was 50 days [standard deviation, SD 66] – nearly 2 months, but the distribution was considerably skewed. The median waiting time was 19.5 days [Interquartile range, IQR 4–60]. The time kept on hold to make the appointment was negligible at about 1 min (63 s, SD = 77) but could take up to ~ 7 min. Two-thirds of dermatologists accepted private, Medicare, and Medicaid insurance (*n* = 228, 66%); a small number accepted only private insurance (*n* = 12, 4%) or no insurance at all (*n* = 16, 5%). The median waiting time for an appointment for the 228 providers that accepted Medicaid was 30.5 days (IQR = 5.0-73.25) while for providers who did not accept Medicaid (*n* = 116) the median wait time for an appointment was 13.0 days (IQR = 3.0–38.0). Just over half (56%) of the dermatologists allowed for appointments to be booked on their website (*n* = 193). This research highlights the necessity of incorporating new strategies into routine dermatology appointments in order to increase treatment availability and decrease healthcare inequality.

## Introduction

In the United States (US), skin cancer is the most commonly diagnosed cancer [1]. Incidence rates of melanoma in the US have increased 320% from 1975 to 2018 [2] and, in New York City (NYC), incidence has risen 250% between 1976 and 2019 from 395/100,000 to 1003/100,000 [3]. It is estimated that 1 in 5 Americans will develop some form of skin cancer by the age of 70 [4].

The majority of skin cancers, including melanoma, are caused by exposure to the harmful ultraviolet (UV) radiation of the sun that damages DNA in the skin and causes abnormal cells to form [5]. Indoor tanning poses a greater skin cancer risk, particularly for women [6], by emitting an amount of UV radiation about 10 to 15 times greater than the sun at its peak intensity [7] and is linked to 6,200 cases of melanoma each year [8]. NYC has a high concentration of indoor tanning facilities, predominantly in the more affluent borough of Manhattan, strategically located to maximize patronage [9] among young, non-Hispanic White women [10, 11].

Across all stages of melanoma, the average five-year survival rate is 94% in the US but when detected early, survival is 99%; survival rates fall to 74% when the disease involves the lymph nodes and to 35% if the disease has metastasized [12]. Early detection and timeliness of treatment is paramount for optimal melanoma outcomes. One study of the National Cancer Database found that patients diagnosed with stage 1 melanoma between 2004 and 2012 who were treated 30–59 days after a biopsy had a 5% higher risk for mortality compared to patients treated within 30 days [13]. Longer delays in treatment resulted in greater mortality with a 41% greater risk of mortality among patients with stage 1 melanoma who were treated > 119 days after biopsy compared with those who received more timely treatment. Beyond the human cost, the financial burden of melanoma cancer care in the US has increased more than 16% in the span of only 5 years from $4.9 billion dollars in 2015 to $5.7 billion in 2020 [14].

Little is known about patients’ experience accessing dermatologic care. Three published studies were identified: [15–17]. The first examined access by patient insurance type and clinic ownership (private equity vs. non-private equity) at 611 sites across 28 US states [15]. The second evaluated access to care at 216 practices in Ontario, Canada for medical (urgent vs. non-urgent) and cosmetic dermatologic appointments, which showed that substantially longer wait times persisted where there was a lower density of dermatologists [16] The third study assessed access to 10–20 practices in each of 15 metropolitan centers around the US [17]. Collectively, these studies found wait times ranging from 7 days to 12.7 weeks depending on patient insurance type, clinic ownership, and indication for the appointment. None of the studies provide a base of comparison for consumers in NYC, the most populous city in the US, where incidence rates of melanoma have been rising.

Efficient and timely diagnosis is essential for treating melanoma as delayed access is associated with a later stage of diagnoses, and concomitant increased mortality risk and excess healthcare costs [12–14]. As such, the purpose of this cross-sectional study was to examine current wait times for new patients to receive dermatological care in NYC. Data pertaining to the time kept on hold required to schedule an appointment, availability of web-based booking, and insurances accepted by providers were also collected to pinpoint additional determinants of treatment access.

## Methods

### Study setting and design

Websites at 58 accredited private and public hospitals in the five boroughs of NYC were reviewed to identify dermatology practices. We examined wait time by simulating a new patient seeking an appointment [18, 19]. All data were collected by telephone using a structured script.

### Sampling, eligibility criteria, and data collection

**Fig. 1:**
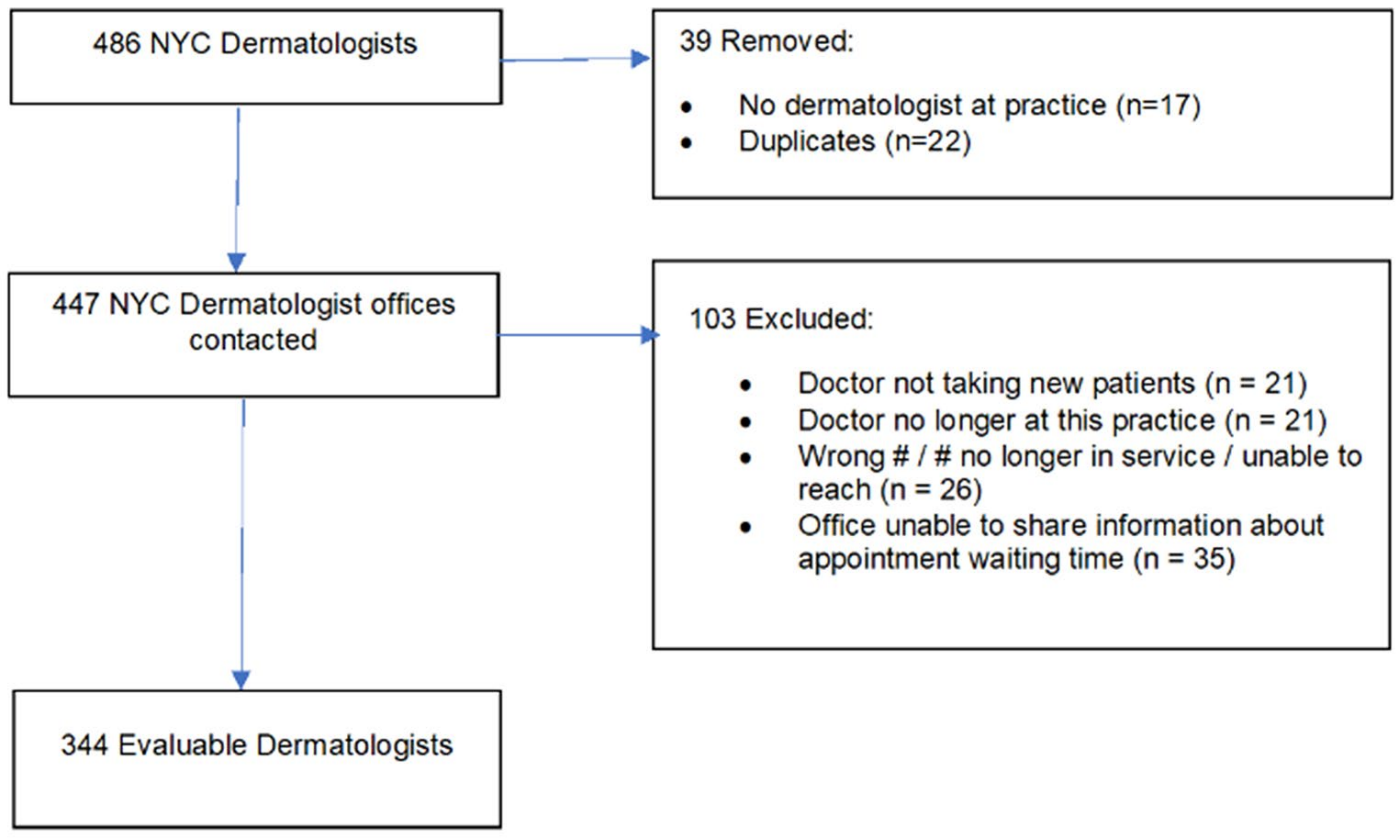
Dermatologists affiliated with NYC hospitals

The office telephone numbers of NYC-based dermatologists listed on each website were called by one author (BG) to collect information pertaining to whether the physician was accepting new patients, type of insurance accepted (public, private, both, or none), and the number of days until a new patient could be seen for an appointment. If prompted for the reason for the visit, the response was, a ‘skin concern.’ Physicians who were no longer working at the practice, who could not be reached (e.g., phone disconnected or out of order), or whose offices could not share information about appointment wait time were excluded from the sample. Hospitals or clinics that did not have dermatologists on staff, as well as duplicate practices for any given doctor, were excluded (Fig. 1). Length of time kept on hold while scheduling appointments and the opportunity to book online (through “ZocDoc” or office website) were also noted.

### Data analysis

Descriptive analyses included calculating frequencies and percentages for variables with dichotomous ‘Yes/No’ response formats and means, standard deviations, medians, and interquartile ranges for variables yielding interval-level data. All analyses were conducted using IBM SPSS version 28 [20].

### Ethical considerations

No personal data was collected nor was any information sought about the individual participants. The Institutional Review Board (IRB) at William Paterson University granted this study a non-human subject determination and the IRB at Teachers College, Columbia University deemed the study exempt.

## Results

Of the 486 dermatologists listed on the websites, 344 (70.5%) met eligibility criteria, had viable contact information, and were included in the sample (Fig. 1). The mean waiting time for an appointment was 50 days [standard deviation, SD 66] – nearly 2 months, but the distribution was considerably skewed. The median waiting time was 19.5 days [Interquartile range, IQR 4–60] (Table 1). The time on hold to make the appointment was negligible at about 1 min (63 s, SD = 77) but could take up to ~ 7 min. Two-thirds of dermatologists accepted private, Medicare, and Medicaid insurance (*n* = 228, 66%); a small number accepted only private insurance (*n* = 12, 4%) or no insurance at all (*n* = 16, 5%). The median waiting time for an appointment for the 228 providers that accepted Medicaid was 30.5 days (IQR = 5–73) while for providers who did not accept Medicaid (*n* = 116) the median wait time for an appointment was 13.0 days (IQR = 3.0–38.0) (data not shown). 56% of dermatologists allowed for appointments to be booked on their website (*n* = 193).

**Table 1:** Characteristics of timeliness of care among New York City dermatologists

	Dermatologists (*n* = 344)
Time until appointment (days)	N (%)
Mean [SD]	50 [66]
Median [IQR]	19.5 [4–60]
Time on hold (seconds)	
Mean [SD]	63 [77]
Range	1-416
Insurance accepted	
Private only	12 (4)
Private & Medicare	88 (26)
Private, Medicare, & Medicaid	228 (66)
None	16 (5)
Appointments available on website	193 (56)

## Discussion

Access to health care is influenced by many factors. Amount of time for an appointment is important in treating diseases that can progress rapidly and have harmful consequences. This is the case for malignant melanoma [21]. We found that one-half of dermatologists had an appointment available within 19.5 days. Yet for 25% of the sample, an appointment would take *≥* 60 days. Given the speed with which malignant melanoma may metastasize this magnitude of delay may result in preventable disease having harmful consequences [21]. There are few studies of wait times for dermatologists and this study provides a preliminary estimate in New York City, a place where there are many medical specialists, including a greater number of dermatologists [22]. Wait times may be considerably longer in other areas. One-third of dermatologists in the sample reportedly did not accept Medicaid insurance or uninsured patients, which may further limit access to timely dermatological care. Treatment delays for skin cancer are significant because of the increased incidence, virulence associated with late-stage diagnosis and associated healthcare costs [12–14].

Other studies evaluating wait times to see a dermatologist yielded mixed results, which may be due to differences in methods. Calling on behalf of a fictitious patients with varying types of insurance coverage using a secret shopper study design, Greadore et al. examined “appointment success” in dermatology clinics across 28 states in the US by ownership of the practice, comparing those owned by a private equity group (*n* = 204) versus those owned by a non-private equity group (*n* = 407) [15]. Median wait times for patients with private insurance and Medicare was 7 days (IQR 2–25 days) but was 13 days (IQR 4–33) for patients with Medicaid; clinic ownership was not associated with wait times. Another study, conducted in Ontario, Canada, examined wait times for urgent and non-urgent dermatological care versus cosmetic dermatologic care at 216 practice locations [16]. Requests for cosmetic appointments had the shortest wait time (3 weeks, IQR 0.4–3.4 weeks) versus 9.0 weeks for urgent medical (IQR = 2.1 to 12.9 weeks) and 12.7 weeks for non-urgent medical care (IQR = 4.4 to 16.4 weeks).

A report generated by AMN Healthcare/Merritt Hawkins examined average wait times for new patients seeking care from a variety of specialties including dermatology in 2022 [17]. The survey was conducted by telephone to 10 to 20 offices per specialty in 15 major U.S. metropolitan areas to access time to the next available appointment and to determine whether Medicare and/or Medicaid was accepted by the provider. For dermatologists the average wait time was found to be 23 days. In that report, 55% of the dermatologists accepted Medicare and 27% accepted Medicaid insurance.

Emerging developments in machine learning and artificial intelligence have expanded opportunities for remote evaluation of skin cancer [22–25], which can help mitigate delays in accessing care. Multiple studies demonstrate the promise of tele-dermatology [26–28], but additional research is needed to improve their application on a widespread basis. As technology continues to improve, innovations that expand remote access to screening and diagnosis are promising ways to reduce wait times.

Individuals of lower socioeconomic status are likely to be disproportionately affected by delayed appointments. One-third of dermatologists in our sample reportedly did not accept Medicaid insurance or uninsured patients, which may further limit access to timely dermatological care. Indeed, compared with providers who did not accept Medicaid, the median wait time for providers who did accept Medicaid was more than twice as long (30.5 days versus 13.0 days). This finding is consistent with previous studies suggesting that, compared with individuals with private insurance, those with Medicaid are 3.3 times less likely to successfully schedule specialty care appointments, as providers were significantly less likely to accept Medicaid due to low reimbursement rates [29]. This may contribute to worse skin cancer outcomes among individuals with lower income [30] and reflects how socioeconomic status remains a key determinant in access to healthcare [31] and health disparities [32].

The geographic scope of this study was limited to NYC, where the concentration of clinical specialists is much greater than in other areas [33]; as such, it is possible that appointment wait times outside of NYC are longer than we observed. This study was also limited by the fact that many medical office websites contained outdated information regarding physician employment at the office, appointment availability, and contact information. However, this finding highlights an additional barrier to appointment accessibility, which likely leads to patient confusion and frustration. Lastly, the cross-sectional nature of this study limits generalizability over time.

## Conclusions

Overall, our research highlights the necessity of incorporating new strategies, potentially through telehealth and AI, into routine dermatology appointments in order to increase treatment availability and decrease healthcare inequality.

## Data Availability

No datasets were generated or analysed during the current study.
